# Temperature Dependent on Mechanical and Rheological Properties of EPDM-Based Magnetorheological Elastomers Using Silica Nanoparticles

**DOI:** 10.3390/ma15072556

**Published:** 2022-03-31

**Authors:** Rusila Zamani Abdul Rashid, Nurul Azhani Yunus, Saiful Amri Mazlan, Norhasnidawani Johari, Siti Aishah Abdul Aziz, Nur Azmah Nordin, Muntaz Hana Ahmad Khairi, Mohd Aidy Faizal Johari

**Affiliations:** 1 Engineering Materials & Structures (eMast) iKhoza, Malaysia-Japan International Institute of Technology (MJIIT), Universiti Teknologi Malaysia, Kuala Lumpur 54100, Malaysia; rusilazamani21@gmail.com (R.Z.A.R.); norhasnidawani@utm.my (N.J.); aishah118@gmail.com (S.A.A.A.); nurazmah.nordin@utm.my (N.A.N.); hana5700@gmail.com (M.H.A.K.); mohdaidyfaizal@graduate.utm.my (M.A.F.J.); 2 Mechanical Engineering Department, Universiti Teknologi PETRONAS, Seri Iskandar 32610, Malaysia

**Keywords:** magnetorheological elastomer, ethylene propylene diene monomer, silica, temperature, rheological properties

## Abstract

Temperature is one of the most influential factors affecting the performance of elastomer matrix in magnetorheological elastomer (MRE). Previous studies have utilized silica as a reinforcing filler in polymer composite and as a coating material in MRE to improve the thermal stability of the base material. However, the usage of silica as an additive in the thermal stability of MRE has not been explored. Thus, in this study, the effect of silica as an additive on the temperature-dependent mechanical and rheological properties of ethylene propylene diene monomer (EPDM)-based MREs was investigated by using 30 wt.% carbonyl iron particles (CIPs) as the main filler, with different contents of silica nanoparticles (0 to 11 wt.%). The microstructure analysis was examined by using field-emission scanning electron microscopy (FESEM), while the thermal characterizations were studied by using a thermogravimetric analyzer and differential scanning calorimetry. The tensile properties were conducted by using Instron Universal Testing Machine in the absence of magnetic field at various temperatures. Meanwhile, the rheological properties were analyzed under oscillatory loadings in the influence of magnetic field, using a rotational rheometer at 25 to 65 °C. The results revealed that the temperature has diminished the interfacial interactions between filler and matrix, thus affecting the properties of MRE, where the tensile properties and MR effect decrease with increasing temperature. However, the presence of silica capable improved the thermal stability of EPDM-based MRE by enhancing the interactions between filler and matrix, thus reducing the interfacial defects when under the influence of temperature. Consequently, the incorporation of silica nanoparticles as an additive in EPDM-based MRE requires more exploration, since it has the potential to sustain the properties of MRE devices in a variety of temperature conditions. Thus, the study on the temperature-dependent mechanical and rheological properties of MRE is necessary, particularly regarding its practical applications.

## 1. Introduction

Progressive development in the new era of technology has resulted in numerous productions of intelligent materials, such as the passive behavior revolution of conventional elastomers to the real-time controllable stiffness of magnetorheological elastomers (MREs). These materials are categorized as smart composite materials, which are composed of ferromagnetic particles embedded in an elastomeric matrix, where its rheological properties can be varied by the application of an external magnetic field [1,2,3,4]. In addition, the controllable properties of MREs have attracted wide attention in semi-active vibration control applications, such as vibration isolator [5,6] and vibration absorbers [7,8]. Previous studies have reported that the ideal concentration of magnetic particles in MRE fabrication was about 30 vol.% (~70 wt.%), since, the higher the particles content, the higher the storage modulus and MR effect. However, too high of an amount of particles would lead to the aggregation of particles, and degradation of mechanical properties and durability [9]. Thus, some researchers have manipulated the composition of magnetic particles as low as 10 to 20 vol.% (40 to 60 wt.%) to enhance the mechanical properties of MRE. For example, Perales Martinez et al. [10] and Puente-Cordova et al. [11] used 10 to 40 wt.% carbonyl iron particles (CIPs) to study the mechanical and rheological properties of silicon rubber (SR)-based MRE. Both results were aligned in which stated that the best CIP concentration for mechanical properties was at 20 wt.%. In the meantime, Aziz et al. [12] has introduced 0.1 wt.% of multiwall carbon nanotube (MWCNT) as an additive with 30 wt.% CIP to improve the mechanical properties of natural rubber (NR)-based MRE. The inclusion of nano-sized additive has enhanced the tensile strength by almost 11% through the improvement in the interfacial interactions between filler and matrix. Thus, the findings showed that the optimum mechanical properties of MRE could be achieved by reducing the magnetic particles concentrations as low as 20 wt.%, and the introduction of additive was another alternative to limit the particles loading, but at the same time, the study managed to enhance the mechanical properties of MRE.

Meanwhile, previous studies on the polymer composite [13,14,15,16] have demonstrated that the incorporation of silica has enhanced the mechanical properties due to the strong reinforcement between silica and polymer matrix. Mokhothu et al. [13,14] reported that the presence of 20 wt.% silica nanoparticles has improved the tensile strength of ethylene propylene diene monomer (EPDM) by almost 118%. Another study by Torbati-Fard et al. [15] showed the effect of different reinforcing fillers (silica and carbon black (CB) and modified silica) at constant concentration of 26 wt.% on the mechanical properties of styrene butadiene rubber (SBR). The results revealed that the silane-modified silica has the highest tensile strength (22.22 MPa), followed by the silica filled SBR (10.84 MPa) and CB-filled SBR (10.74 MPa). In fact, the addition of 11 wt.% silica nanoparticles as an additive in the EPDM-based MRE has enhanced the tensile strength by almost 344%, increased from 3.18 to 14.13 MPa [17]. 

Apart of improvement in mechanical properties, it was also reported that the silica could enhance the thermal stability of polymer composite [13,18,19]. The reinforcement of silica has strengthened the interfacial interactions between filler and matrix, thus improving the thermal stability of polymer matrix, since it delayed the degradation of the matrix at a certain temperature. According to Azizi et al. [18], the utilization of 9 wt.% modified fume silica improved the thermal stability of EPDM matrix by 11 °C, increased from 403 to 414 °C. A similar study has been reported by Khan et al. [19], where the nano-sized silica had higher thermal stability of 6 °C than micro-sized silica in EPDM matrix. Meanwhile, Mokhothu et al. [13] have investigated the EPDM reinforced in situ silica with surface treatment with a silane-coupling agent. Their findings showed that the additional of 30 wt.% treated silica nanoparticles has improved the interaction between silica and EPDM by 10 °C in thermal stability from 426 to 436 °C as compared to previous researches with the absence of a coupling agent [14,20]. 

In the meantime, silica has also been recognized as one of the effective coating materials to magnetic particles in order to improve the interfacial interactions between magnetic particles and elastomer matrix in MRE fabrication. According to Malecki et al. [21], the coated CIPs exhibited a higher thermal stability than pure CIPs in the styrene ethylene butylene styrene (SEBS)-based MRE, where it started to decompose from 230 to 300 °C. The strong affinity between CIPs surface and silica has led to a good adhesion between CIPs and matrix, which consequently increased the thermal stability of MRE, as also reported elsewhere [22]. Based on the findings, it could be concluded that the utilization of silica as reinforcing filler and coating material managed to enhance the thermal stability of polymer matrix and MRE, respectively. 

As of now, previous studies have utilized almost 20 to 30 wt.% of CIPs to obtain the optimal mechanical properties of MRE. Moreover, temperature is also considered as one of the most influential factors that affects the properties of elastomer matrix in real MRE applications. Meanwhile, the utilization of silica as reinforcing filler in polymer composite has demonstrated an improvement in mechanical, as well as thermal stability of polymer composite. Despite that, silica has also been implemented as a coating material in MRE to improve the interfacial interactions and thermal stability of the base material. However, the usage of silica as an additive in thermal stability of MRE needs to be performed for further understanding of its influence on temperature. Thus, in this study, the effect of temperature-dependent performance of EPDM-based MREs containing 30 wt.% CIP with various contents of silica nanoparticles, and its correlation with the mechanical and rheological properties is investigated. EPDM was selected due to its high strength, good resistance to temperature, ageing, oxidation and atmosphere, easier processing steps and low production cost compared to NR [9,22]. Furthermore, the utilization of silica nanoparticles as an additive is expected to change the decomposition mechanism of elastomer matrix by improving the interfacial interactions between filler and matrix. Consequently, the thermal stability of EPDM-based MRE under various operating temperatures and magnetic field will be improved.

## 2. Experimental

### 2.1. MRE Fabrication

The EPDM (EPT 4010 Mitsui, Tokyo, Japan) was used as the matrix, provided by Titron Rubber Sdn Bhd. The spherical shape of CIPs (BASF, Ludwigshafen, Germany) was used as magnetic particles with an average particle size of 6 µm and density of 7.8 g/m^3^. Silica in a spherical shape was used as an additive with an average diameter size of 20–25 nm that was obtained from Sky Tech Malaysia Sdn Bhd (Kuala Lumpur, Malaysia). The zinc oxide (ZnO) and stearic acid were acted as vulcanizing activators, while sulfur was used as a vulcanizing agent. In addition, 2-Mercaptobenzothiazole (MBT 80) and tetramethyl thiuram disulfide (TMTD) were utilized as accelerators, and Si69 was used as silane-coupling agents in EPDM-based MRE compositions. Five samples were fabricated, as the silica contents were varied by 0, 3, 6, 9 to 11 wt.% according to the formulation S1–S5, respectively. The mass fraction of CIPs was fixed at 30 wt.% for all samples. The fabrication process of the isotropic EPDM-based MREs involved two steps: mixing and curing. The compounding ingredients were mixed by using double-roll mill for 30 min to the produce final sheet form compound. Afterward, the MRE samples in the form of sheet were cured at 150 °C without the presence of magnetic field for 30 min to ensure the particles were locked into the matrix.

### 2.2. Physicochemical Properties

The microstructure images of cross-section MRE samples were observed by using field-emission scanning electron microscope (FESEM) (Hitachi High-Tech Corporation, Tokyo, Japan) equipped with Energy-Dispersive X-ray, EDX (Oxford Instruments XmaxN, Oxford, UK). For FESEM analysis, a sample size of 10 mm × 10 mm was prepared, coated with 1 mm platinum and examined under voltage of 5 kV with 1.5 and 15 k× magnifications. In addition, the thermogravimetric analyzer (TGA) was carried out by using an STA 8000: Perkin Elmer (Waltham, MA, USA) in the nitrogen atmosphere. The sample was continuously heated from 25 up to 800 °C with the heating rate of 10 °C/min. The glass-transition temperature (T_g_) and melting temperature (T_m_) were studied by differential scanning calorimetry (DSC-60 apparatus, Shimadzu, Japan) under nitrogen atmosphere. The sample was cooled from ambient temperature to −80 °C, followed by heating up to 100 °C at 10 °C/min heating and cooling rate. Meanwhile, the tensile strength and elongation at break of MRE samples were obtained by using a universal testing machine (AG-X Series by Shimadzu, Kyoto, Japan) with a crosshead speed of 500 mm/min at 25, 50, 75, 100 and 125 °C, according to ASTM D412 standard. The cured MRE samples were prepared in “dog bones” geometry with a dimension of approximately 2 mm × 5 mm with 20 mm gauge length, and the average measurement of three samples was reported for samples S1, S3 and S5 that contained 0, 6 and 11 wt.% silica, respectively.

### 2.3. Rheological Properties

The rheological properties of EPDM-based MRE samples were measured by using an advanced commercial rheometer (Physica MCR 302, Anton Paar, Graz, Austria). The rheometer was equipped with magneto-controllable accessory (MRD 70/1T) and profiled parallel plate measuring system (PP20/MRD/T1/P2). A temperature-control device (Viscotherm VT2, Anton Paar, Graz, Austria) was used to adjust the measuring temperature. A sample with a diameter of 20 mm diameter and 1 mm thickness was placed between rotating disk and a parallel base plate. The magnetic field strength was generated by the controlled current applied to the electromagnetic coil and recorded by using a Teslameter (Anton Paar, Graz, Austria), where the magnetic field strength was parallel to the thickness of the sample. A pre-force of 5 N was applied to all MRE samples to prevent the sample from sliding on the oscillating disk. The magnetic flux density values were generated by varying the controllable input currents from 0, 1, 2, 3, 4 and 5 A, which are equivalent to 0, 0.18, 0.36, 0.53, 0.69 and 0.8 T, respectively. The test was conducted in a shear oscillating mode to investigate the rheological properties of EPDM-based MRE for current and temperature sweep tests. For the temperature sweep test, the effect of storage modulus was investigated at different temperatures of 25, 35, 45, 55 and 65 °C under a constant magnetic field (0.2, 0.4, 0.6 and 0.8 T). Moreover, the samples were also tested under different magnetic flux densities from 0 to 0.8 T with constant frequency (1 Hz) and strain amplitude (0.01%) for various temperatures (25 to 65 °C).

## 3. Results and Discussion

### 3.1. Microstructure Appearance

**Figure 1:**
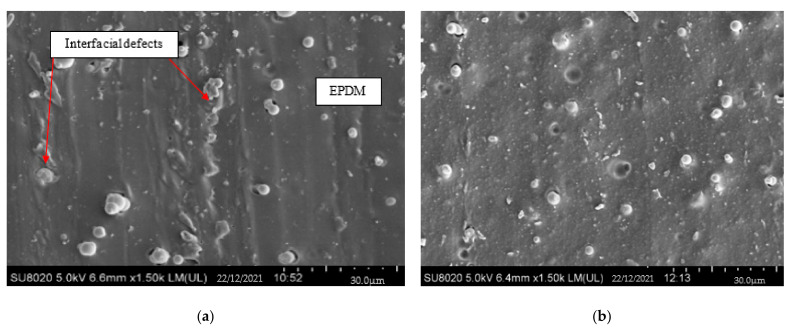

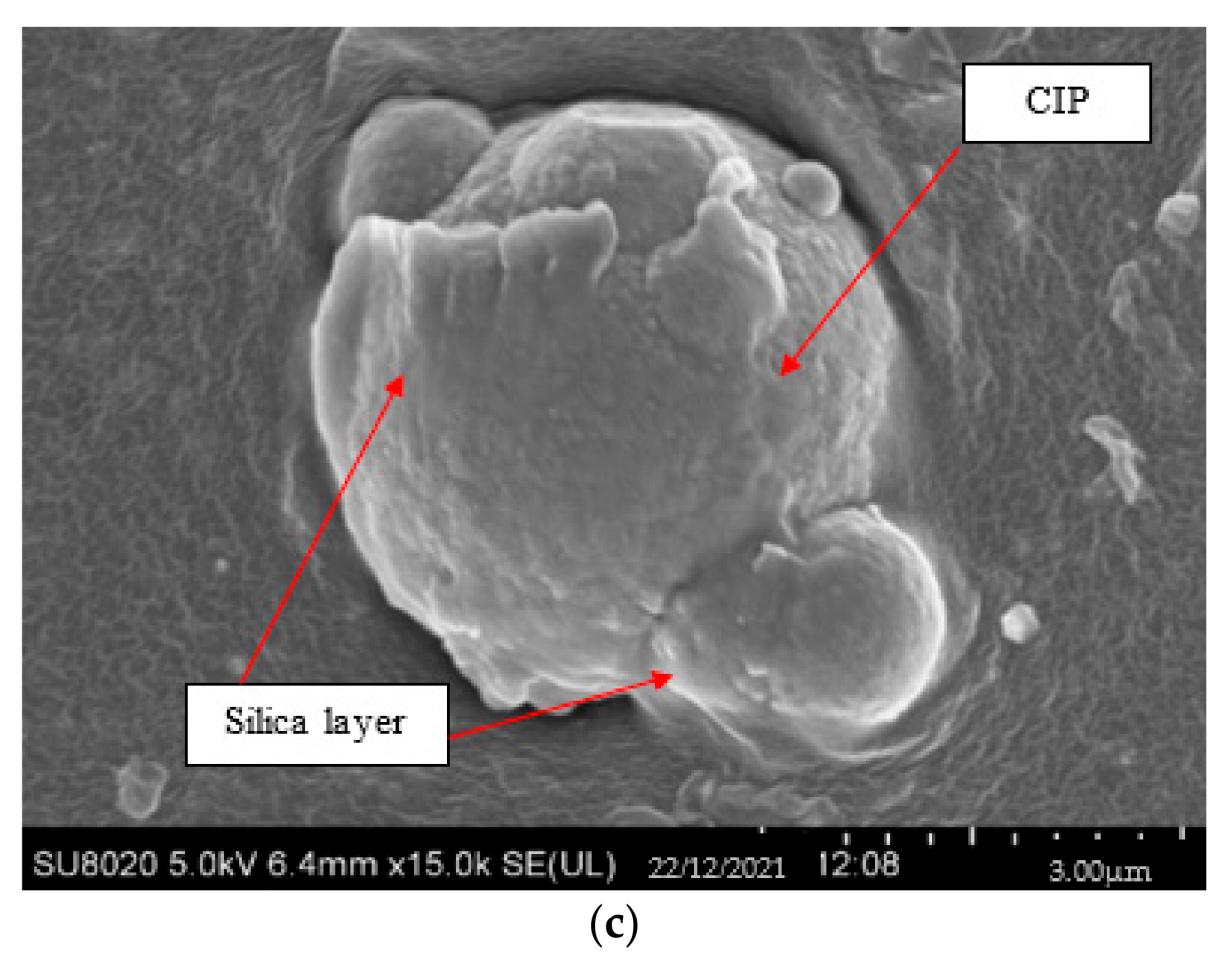
FESEM micrograph of EPDM-based MRE: (**a**) sample S1 (0 wt.% silica), sample S5 (11 wt.% silica) at (**b**) 1.5 k× and (**c**) 15 k× magnification.

The FESEM images of cross-section EPDM-based MREs for sample S1 (0 wt.% silica) and sample S5 (11 wt.% silica) after rheological test under temperature are shown in Figure 1.

As shown in Figure 1a, it can be observed that the CIPs are randomly distributed, with some of them embedded in the EPDM. Some unique phenomenon was observed in S1, as the interfacial defects clearly appeared after the temperature test [22]. Meanwhile, in S5 (Figure 1b), it is clearly observed that the CIPs and silica are embedded in the EPDM matrix, indicating a positive interfacial interactions between fillers and matrix [17]. In the meantime, some of the silica is adhered on the surface of CIPs and could be seen as a layer on the surface of CIP as seen in Figure 1c. Basically, when the polymer is exposed to a high temperature (>50 °C), the molecules in the form of long polymer chains would vibrate and stretch, causing the chains to disentangle and slide more easily. Thus, the polymer chains are diminished, resulting in interfacial defects between filler and matrix. Nevertheless, in S5, the minimum interfacial defect was observed, as the presence of silica restrained the mobility of matrix molecular chains and might prevent the molecular chains of EPDM matrix from being diminished due to the temperature influence [13,23]. Thus, this microstructure observed in this sample has proved that the silica might protected the matrix from being affected by heat and minimum interfacial defects was seen in MRE as compared to Figure 1a without the presence of silica.

**Figure 2:**
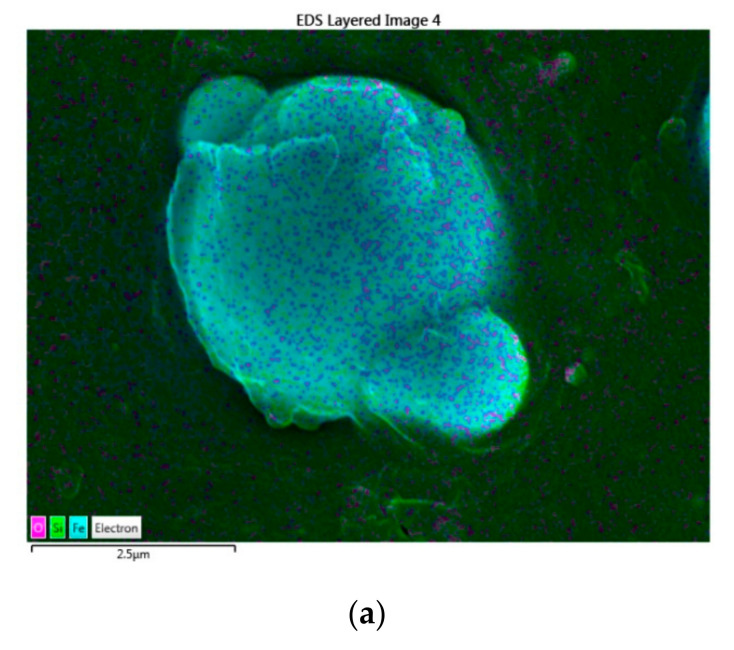

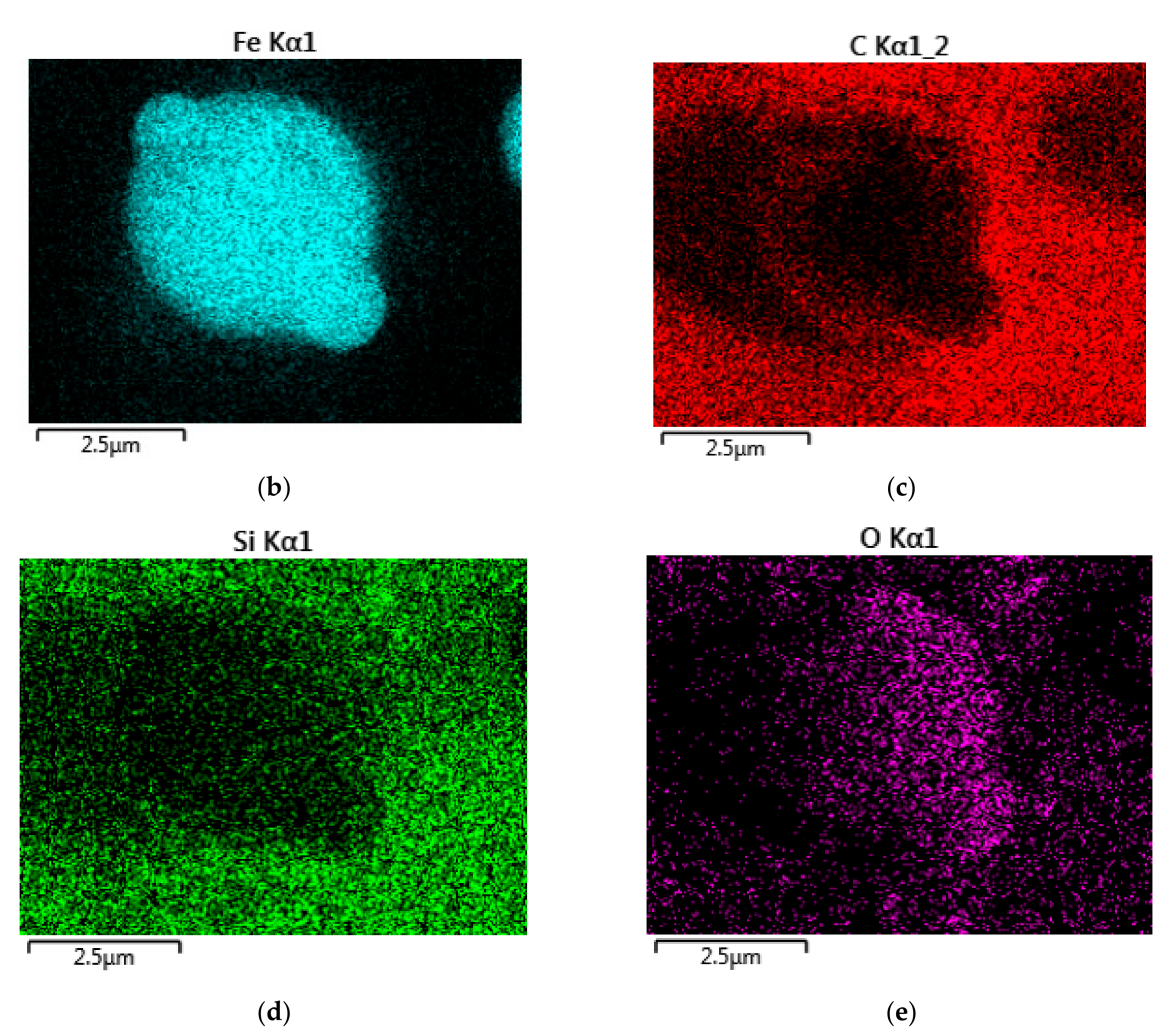
EDX mapping analysis of EPDM-based MRE sample with 11 wt.% silica consisting of (**a**) EDX region, (**b**) iron (Fe), (**c**) carbon (C), (**d**) silicone (Si) and (**e**) oxygen (O) elements.

The distribution of silica and CIPs within the EPDM is presented in Figure 2 through the EDX mapping analysis.

The CIPs that were represented by the element Fe and the silica (SiO_2_), which is composed of Si and O elements, were observed throughout the MRE. Therefore, the mapping results were aligned with FESEM microstructure observations, where the spherical shape represented the CIPs and some of the silica particles were observed on the surface of CIPs.

### 3.2. Thermal Stability

#### 3.2.1. Thermogravimetric Analysis (TGA)

**Figure 3:**
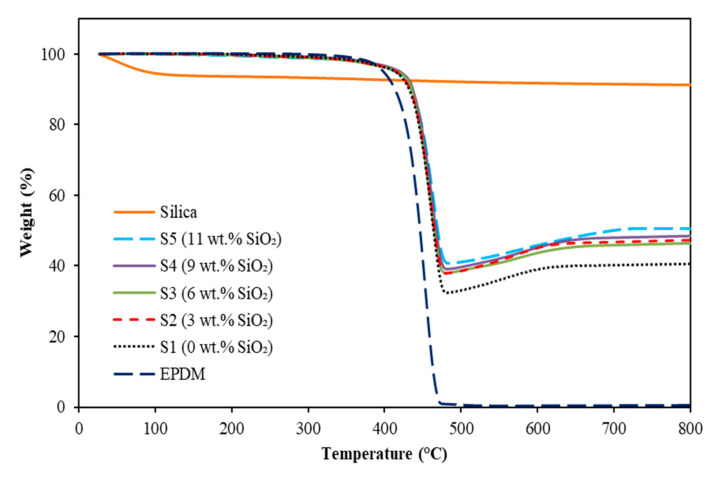
TGA curves of EPDM silica and EPDM-based MREs with different contents of silica.

The effect of silica on the thermal properties of EPDM-based MREs was investigated by thermogravimetric analyzer (TGA), and the results are shown in Figure 3.

The thermogravimetric (TG) curves of EPDM, silica and EPDM-based MREs with different contents of silica were analyzed by comparing the weight loss as a function of temperature, while the characteristics of thermal decomposition are demonstrated in Table 1.

The thermal stabilities of MRE were characterized in terms of temperatures taken at the onset (T_onset_), which refers to the beginning of the weight loss and at 50% weight loss (T_50%_). Figure 2 shows no change in weight for the pure silica during the thermal decomposition analysis. Meanwhile, the weight of EPDM matrix was constant up to 400 °C before dropping drastically from 426 to 470 °C. On the other hand, the MRE samples indicated almost similar trends as EPDM matrix; however, the decomposition of MRE samples started to drop slightly later at around 430 to 439 °C. The MRE sample with 0 wt.% silica started to decompose around 430.3 °C, and no change in weight was seen beyond the temperature of 463.9 °C. Nevertheless, the addition of 3 and 6 wt.% (samples S2 and S3) depicted some minor effect (almost 5 to 6 °C) in thermal stability of EPDM-based MRE. In contrast, MRE was having a higher thermal stability at a higher content of silica, as indicated by S1, S4 and S5 at 430.3, 437.7 and 438.5 °C, respectively. This might be due to the strongly bound and well-dispersed silica nanoparticles in MRE that restrained the mobility of matrix molecular chains and retarded the diffusion of volatile products, thus delaying the decomposition of EPDM in MRE [13,24]. In addition, the improved interfacial interactions between CIPs and matrix by the adhesiveness of silica on the surface of the CIPs has reduced the degradation at a certain temperature, as well as the maximum degradation rate of the EPDM. This finding agrees well with Aziz et al. [12], as the presence of MWCNT particles in MRE has restricted the movement in polymer macromolecules chains, resulting in the changes in decomposition mechanism of MRE. The above finding was also complied with the finding by Nayak et al. [25], which revealed that the addition of carbon black (CB) has attenuated the time rate of weight loss. Thus, it can be concluded that the addition of nanoparticle or additives that possessed good thermal properties in MREs will significantly affected the thermal stability of MREs as increasing the reaction time.

**Figure 4:**
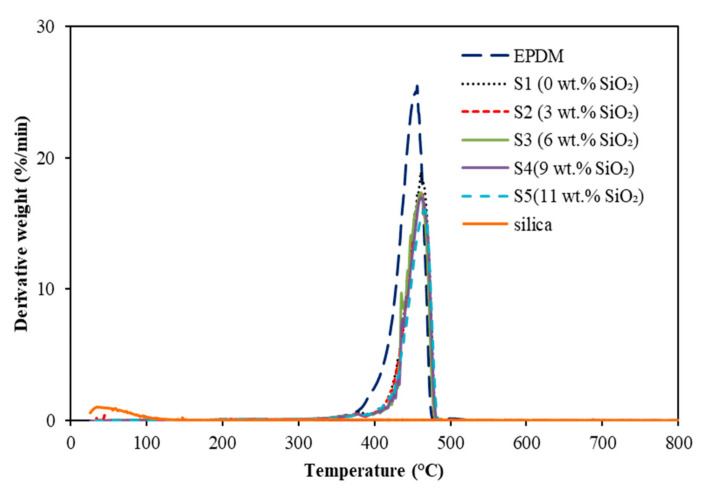
DTG curves of EPDM, silica and EPDM-based MREs with different contents of silica.

Meanwhile, the temperature of maximum decomposition rate of EPDM-based MREs is represented in the Derivative Thermogravimetry (DTG) curves, as illustrated in Figure 4.

A similar temperature region was revealed in DTG curves as in the thermal degradation in TG curves, which was in the range of 400 to 475 °C. The peak decomposition temperature, T_p_, for the EPDM matrix was around 464.3 °C, while no peak was detected for silica particle. Instead, the addition of silica has significantly improved the thermal stability of EPDM-based MRE, where T_p_ of sample containing 11 wt.% silica shifted toward the higher temperature of 473.5 °C. However, insignificant changes were observed between the MRE samples containing silica for the T_p_ results, as listed in Table 1.

#### 3.2.2. Differential Scanning Calorimetry (DSC)

**Figure 5:**
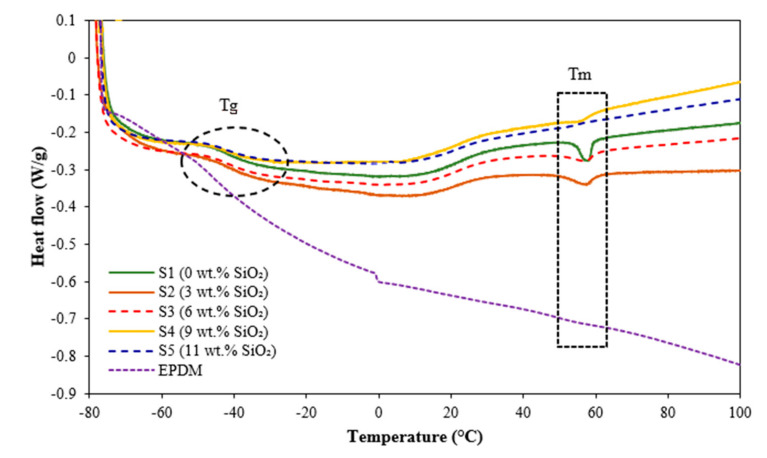
DSC curves of neat EPDM and EPDM-based MREs with different contents of silica.

The influence of temperature on the thermal characteristics of the neat EPDM and EPDM-based MREs at different silica contents was analyzed by using differential scanning calorimetry (DSC), as illustrated in Figure 5, while the thermal behaviors of all samples are demonstrated in Table 2.

Figure 5 shows an almost similar trend in glass-transition temperature (T_g_) for all MRE samples, where the T_g_ value shifted toward a high temperature with the increment of silica content due to the phase transformation from glassy to rubbery state. From the result obtained in this study, the T_g_ value of the neat EPDM is −43.7 °C and further increased from −42.63 to −39.95 °C, following the increment of silica from 0 to 11 wt.%. The addition of silica has restricted the mobility of the polymer molecular chain in an MRE and, thus, shifted the T_g_ value to higher values. This finding agreed well with previous studies [26,27] in which the T_g_ value was slightly increased by the addition of silica in polymer matrix.

Meanwhile, during heating process below 100 °C, the endothermic peak of neat EPDM was not observed, as the melting temperature (T_m_) of EPDM is normally started at temperature > 150 °C [28]. However, the endothermic peak was observed with the presence of particles (CIP and silica) in the EPDM-based MREs. The endothermic peaks were clearly observed in the samples S1, S2 and S3 at around 58 °C, where the T_m_ slightly decreased with the increment of silica at 57.94, 57.28 and 57.08 °C, respectively. This finding might be related to the onset of molecular motion in the crystalline phase or known as alpha-phase transition of the particles-reinforced EPDM matrix [29,30]. Nonetheless, as for samples S4 and S5, the endothermic peaks were almost invisible and the T_m_ of EPDM-based MRE samples was lower compared to S1, S2 and S3. The finding is parallel to the decrement of ΔH_m_ from 1.08 to 0.68 J/g as the silica content increased as shown in Table 2. The result is correlated with the study by Kim et al. [26] which has reported a similar trend, where the incorporation of silica nanoparticles has slightly decreased the T_m_ and ΔH_m_ of poly(ethylene 2.6-naphthalate) (PEN)/silica hybrid nanocomposite. The higher content of silica in MRE would act as a thermal resistance to reduce the heat absorption by the CIPs in the MRE samples [31,32,33]. Thus, it can be concluded that the addition of silica could decrease the crystallinity of EPDM matrix in MRE.

### 3.3. Tensile Properties

**Figure 6:**
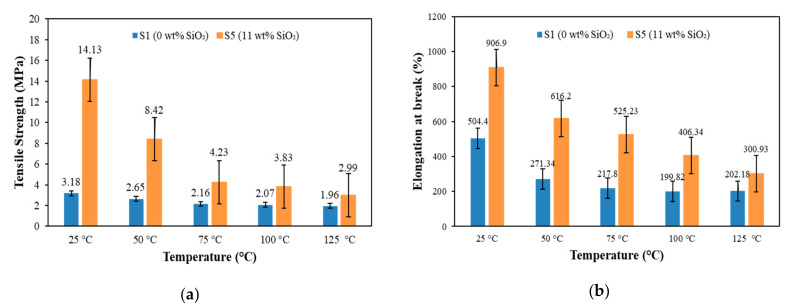
Tensile properties of EPDM-based MREs: (**a**) tensile strength and (**b**) elongation at break at different temperatures for sample S1 and S5.

Figure 6 shows the effect of temperature on the tensile strength and elongation at break for sample with and without silica. For both samples, the results revealed that the tensile strength decreased with increasing temperature, as presented in Figure 6a.

Initially, the tensile strength of the sample without silica (sample S1) decreased rapidly from 3.18 to 2.16 MPa with the increase of temperature from 25 to 75 °C. Then the tensile strength was further decreased slowly until 1.96 MPa at 125 °C. A similar trend was also observed in the sample containing 11 wt.% silica (sample S5), where the tensile strength was obviously seen in two continuous patterns. The tensile strength decreased rapidly from 14.13 to 4.23 MPa when the temperature increased from 25 to 75 °C and then continued to decrease slowly until 2.99 MPa at 125 °C. This behavior mainly related to the effect of temperature, which diminished the molecular chains of EPDM, resulting in interfacial defects between the filler and matrix [22,34], as shown in Figure 1a. The weak interfacial interaction between fillers (CIP and silica) and EPDM disrupted the stress transfer between fillers and matrix, causing a decrement in tensile strength of MRE. Moreover, the molecular chains of EPDM have further reduced after exceeding the melting temperature of EPDM matrix (almost 60 °C), as reported in DSC analysis. Consequently, more interfacial defects between filler and matrix were formed and led to a slow decrement in tensile strength. This result is supported by Hussein et al. [34], where the tensile strength decreased with increasing temperature; however, the decrement was becoming slower after the melting temperature.

Meanwhile, a similar trend of elongation at break can be observed in Figure 6b, where the elongation at break of sample S1 decreased rapidly from 504.4 to 217.34% with the increased of temperature from 25 to 75 °C. After that, the elongation at break was further decreased gradually until 217.8% at 100 °C before reaching saturation value at 125 °C. Nevertheless, the elongation at break for sample S5 rapidly decreased from 906.9% to 300.93% when the temperature increased from 25 to 125 °C. In general, the heat application disrupted the molecular structure of elastomer matrix in the MRE [30,35], hence resulting in a weak adhesion between fillers (CIP and silica) and matrix due to interfacial defects, as revealed in Figure 1a, causing a decrement in the elasticity of EPDM-based MRE. However, the presence of silica has improved the adhesion between filler and matrix and caused stress transfer between them, resulting in a better interaction on the surface of MRE, even under the influence of temperature.

**Figure 7:**
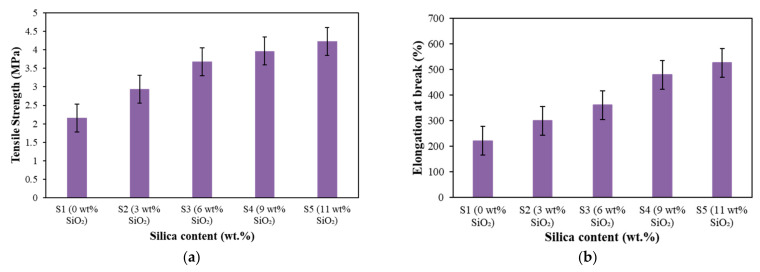
Tensile properties of EPDM-based MREs: (**a**) tensile strength and (**b**) elongation at break at a constant temperature (75 °C) with different silica contents.

On the other hand, a better understanding of the effect of silica on the tensile properties under the application of temperature (75 °C) is shown in Figure 7.

It can be observed that the tensile strength increased with increasing silica contents, as presented in Figure 7a. The tensile strength increased slightly with the increasing silica content, where the values of tensile strength were 2.16, 2.94, 3.68, 3.97 and 4.23 MPa for the silica content of 0, 3, 6, 9 and 11 wt.%, respectively. These results were due to the reinforcement of silica that has strengthened the MRE sample via the improvement in filler-matrix interactions, and simultaneously restrained the movement of molecular chains of the matrix. Consequently, the decomposition mechanism of the elastomer matrix has been reformed through the increment in network chain density of MRE upon heating, as proven by TGA analysis in Figure 3, resulting in an increment of the tensile strength.

Furthermore, it can be seen that the elongations at break are also increased with increasing silica content, as shown in Figure 7b. The values of elongation at break for the addition of 0, 3, 6, 9 and 11 wt.% were at 219.98%, 299.12%, 360.09%, 478.42% and 525.23%, respectively. This finding was mainly related to the adhesiveness of silica into CIPs that has improved the interfacial adhesion between fillers and matrix and enhanced the elasticity of the MRE [17]. The improved adhesion between fillers (CIPs and silica) and matrix has led to a better interaction on the surface of MRE because of the stress transfer between them. As a result, the elongation behavior of the EPDM-based MREs increased even under the influence of temperature.

### 3.4. Rheological Properties

#### 3.4.1. Storage Modulus Variation with Magnetic Field Strength and Temperature

**Figure 8:**
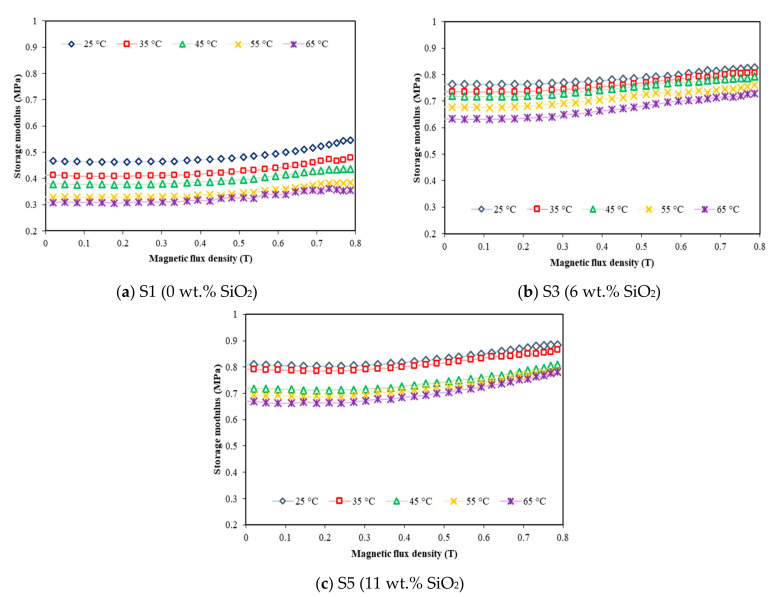
Storage modulus versus magnetic flux density at different temperatures and silica contents of (**a**) 0 wt.%, (**b**) 6 wt.% and (**c**) 11 wt.%.

Figure 8 represents the storage modulus of EPDM-based MREs as a function of the magnetic field at different temperatures for samples containing 0, 6 and 11 wt.% silica (S1, S3 and S5). The results showed that the storage modulus of all samples increased with increasing of the magnetic field up to 0.8 T when the samples were subjected to a constant temperature.

As in the absence of the magnetic field, the initial storage modulus of the sample without silica (Figure 8a) decreased from 0.47 to 0.31 MPa with increasing temperature from 25 to 65 °C. Similar trends were also observed in other samples (S3 and S5) containing 6 and 11 wt.% silica (Figure 8b,c), respectively. The value of initial storage modulus decreased from 0.76 to 0.63 MPa for sample S3, and 0.81 to 0.67 MPa for sample S5 when the temperature increased from 25 to 65 °C. It is noted that the temperature has a great influence on the initial storage modulus of MRE, particularly in the absence of magnetic field, which is mainly decided by the matrix modulus. This phenomenon was a common behavior for polymers in which as the temperature increased; the thermal motion of molecular chain in the polymer was also increased and slowly restricted the interaction between the molecules [35,36]. Thus, the relative movement between molecular chains would cause a continuous decrease in the overall modulus of the elastomer matrix. Meanwhile, the temperature-dependent storage modulus under the presence of magnetic field exhibited almost similar trends for all samples. It was apparent that the storage modulus was increased with the increasing magnetic field, meaning that the MREs were stiffer by increasing the magnetic field strength.

On the other hand, a better understanding about the effect of silica on the MR effect under various temperatures is shown in Table 3. The relative MR effect is evaluated by Equation (1) below:

(1) $$\frac{G_{max}-G_{0}}{G_{0}}\times 100\%,$$

It can be seen that the MR effect of sample S1 decreased from 17.02 to 12.9% as the temperature increased from 25 to 65 °C. This finding is similar with other previous studies [30,35,36,37], and this might be due to the effect of temperature that caused the matrix to become soft, thus causing the interfacial defects between filler and matrix, as shown in Figure 1a. Consequently, the movement of CIPs within the MRE under the application of magnetic field were affected due to the difficulties in the stress transfer between them, thereby contributing to the decrement in the MR effect of MRE. However, the addition of silica enhanced the MR effect of MRE, where the values increased from 7.89 to 14.29% for sample S3 and 8.64 to 16.42% for sample S5 with the increasing of temperature. This finding might be attributed to the adhesiveness of silica into CIPs that filled the gaps between the filler and matrix (Figure 1b,c), resulting in strong magnetic forces between CIPs under magnetic field applied. Therefore, the CIPs were more sensitive toward the change of magnetic field and resulted in the effective stress transfer within the MRE even under the influence of temperature.

#### 3.4.2. Storage Modulus Variation with Various Temperatures

**Figure 9:**
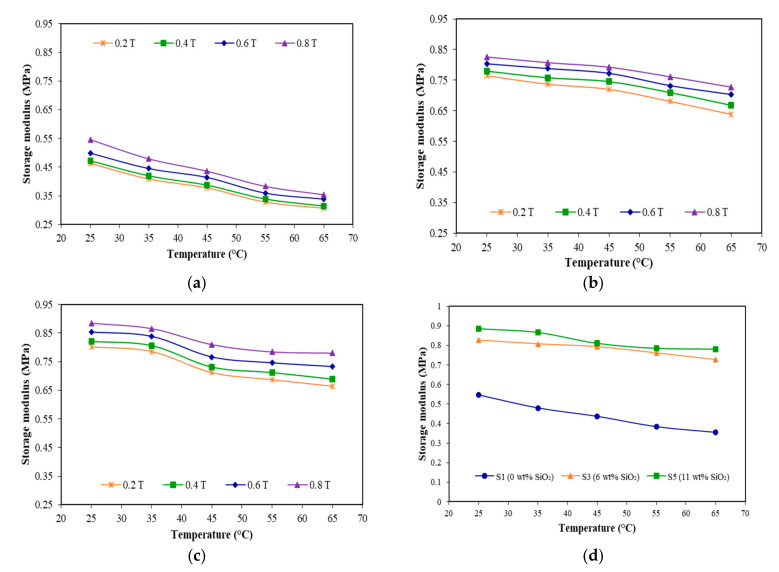
Storage modulus versus temperature at different magnetic fields and silica contents of (**a**) S1 (0 wt.% SiO_2_), (**b**) S3 (6 wt.% SiO_2_) and (**c**) S5 (11 wt.% SiO_2_), and (**d**) comparison of storage modulus versus temperature for all samples at 0.8 T.

The temperatures dependent on the storage modulus under different magnetic fields (0.2, 0.4, 0.6 and 0.8 T) for various silica contents (0, 6 and 11 wt.%) are demonstrated in Figure 9.

As shown in Figure 9, it can be seen that the storage modulus of all samples decreased with the increasing of temperature but tended to increase with the influence of magnetic field and addition of silica. As such, at the low magnetic field of 0.2 T, the storage modulus of sample S1 (Figure 9a) decreased from 0.46 to 0.31 MPa as the temperature rose from 25 to 65 °C, with decrement of 0.15 MPa. Meanwhile, at the high magnetic field of 0.8 T, the storage modulus decreased by 0.20 MPa from 0.55 to 0.35 MPa, and the decrement was larger compared to when the magnetic field was at 0.2 T. Under the application of magnetic field, the magnetic particles were rapidly magnetized and the magnetic interaction between the particles would cause the particles to align accordingly to the direction of external magnetic field, resulting in the increment of storage modulus [35,37]. Meanwhile, under the influence of temperature, the modulus of elastomer matrix would significantly be reduced as the molecular chains were disentangled and led to the interfacial defects between filler and matrix, as revealed in Figure 1a. In relation to this result, the decrement in initial storage modulus is shown in Figure 8. Moreover, under the effect of temperature with increasing of magnetic field, the magnetization of MRE would remarkably diminish and led to the decrement in the storage modulus of MRE as obtained from sample S1.

On the other hand, the presence of silica has exhibited a different trend in which the larger the magnetic field, the smaller the decrement in storage modulus as the temperature increased. The storage modulus of sample S3 (Figure 9b) decreased by 0.13 MPa from 0.76 to 0.63 MPa under the application of low magnetic field (0.2 T). When the magnetic field increased up to 0.8 T, the storage modulus decreased from 0.83 to 0.73 MPa (0.1 MPa decrement). Similarly, the addition of 11 wt.% silica (Figure 9c) during the presence of low magnetic field (0.2 T) decreased the storage modulus from 0.8 to 0.66 MPa (0.14 MPa decrement) as the temperature increased from 25 to 65 °C. Meanwhile, as the magnetic field increased up to 0.8 T, the storage modulus decreased from 0.88 to 0.78 MPa, almost 0.1 MPa decrement in storage modulus with increasing temperature. This finding might be due to the effect of silica that adheres on the surface of CIPs and functions to fill the gaps between filler and matrix, thus reducing the interfacial defects under the application of temperature, as revealed in Figure 1b. Thus, the magnetization of MRE was not much affected and the CIPs were more sensitive toward the change of magnetic field even under the influence of temperature, reducing the decrement in storage modulus of MRE.

In addition, a better understanding on the effect of silica on the storage modulus under various temperatures can also be observed in Figure 9d. It was apparent that the storage modulus decreased gradually with the increasing of temperature, while it increased with the addition of silica. However, it can be seen that the presence of a higher content of silica (for example for sample S5) exhibited a distinct trend, where the storage modulus was initially decreased gradually until 55 °C, before reaching the saturation value at 65 °C. This finding is similar with other previous studies [30,35,36,37], and this might be due to the effect of temperature that caused the matrix became soft, thus effecting the movement of particles within the MRE under the applied magnetic field. In addition, the transition temperature of EPDM matrix, which appeared approximately at 60 °C in DSC analysis in Figure 5, has significantly affected the storage modulus of MRE that has also been reported in several studies [30,36,37].

## 4. Conclusions

In this study, the effect of different concentrations of silica nanoparticles (0 to 11 wt.%) on the temperature dependent mechanical and rheological properties of EPDM-based MRE was investigated. The microstructures, thermal, mechanical and rheological properties of the MRE samples were investigated and discussed thoroughly. Based on the microstructural analysis, we saw that both particles (CIPs and silica) have homogeneously disseminated within the MRE and some of them were embedded in EPDM. Moreover, the presence of silica has protected the matrix from being affected by the temperature, as the interfacial defects were almost visible in the MRE sample. The thermal characterization test showed that the incorporation of silica improved the thermal stability of MRE due to an increment in the thermal decomposition temperature from 209.8 to 234.6 °C. In the meantime, the mechanical properties of MRE were significantly affected by the temperature, as the tensile strength and elongation at break were decreased with increasing temperature. However, the samples’ containing silica has better mechanical properties even under the influence of various operating temperatures. The effect of temperature on the rheological properties indicated that the storage modulus decreased with increasing temperature. Nevertheless, the addition of silica in MRE sample has significantly improved the MR effect from 8.64 to 16.42% with increasing temperature from 25 to 65 °C. Thus, it can be concluded that the incorporation of silica has significantly improved the mechanical and rheological properties of MRE under the influence of temperatures, and this has the potential to be further explored in MRE devices under different operating temperatures.

## Figures and Tables

**Table 1:** Thermal decomposition temperatures of EPDM, silica and EPDM-based MRE samples.

Sample	T_onset_ (°C)	T_50%_ (°C)	T_p_ (°C)	Residue (%)
EPDM	426.3	454.6	464.3	0.891
S1 (0 wt.% SiO_2_)	430.3	463.9	468.5	41.09
S2 (3 wt.% SiO_2_)	434.9	464.8	469.8	48.06
S3 (6 wt.% SiO_2_)	435.8	465.1	471.1	48.3
S4 (9 wt.% SiO_2_)	437.7	467.4	472.7	49.17
S5 (11 wt.% SiO_2_)	438.5	469.5	473.5	50.68

**Table 2:** Thermal behavior of neat EPDM and EPDM-based MRE samples.

Samples	Glass Transition Temperature, T_g_ (°C)	Melting Temperature, T_m_ (°C)	Enthalpy of Fusion, ΔH_m_ (J/g)
S1 (0 wt.% SiO_2_)	−42.63	57.94	1.08
S2 (3 wt.% SiO_2_)	−42.48	57.28	1.01
S3 (6 wt.% SiO_2_)	−42.14	57.08	0.81
S4 (9 wt.% SiO_2_)	−39.97	56.16	0.68
S5 (11 wt.% SiO_2_)	−39.95	-	-
EPDM	−43.7	-	-

**Table 3:** The relative MR effect of EPDM-based MRE for various silica contents under different temperatures.

Samples	Temperature (°C)	Relative MR Effect (%)	
S1 (0 wt.% silica)	25	17.02	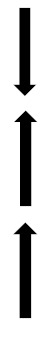
	35	17.07	
	45	15.79	
	55	15.15	
	65	12.9	
S3 (6 wt.% silica)	25	7.89	
	35	9.59	
	45	9.72	
	55	11.76	
	65	14.29	
S5 (11 wt.% silica)	25	8.64	
	35	10.12	
	45	12.5	
	55	13.04	
	65	16.42	

Arrows denote the increment and decrement of MR effect.

## Data Availability

The raw/processed data required to reproduce these findings cannot be shared at this time as the data also part of an ongoing study.
